# Tuning the electronic structure of a rod-like DNA-stabilized silver nanocluster Ag_28_Cl_2_ for photophysics in the NIR-II window†

**DOI:** 10.1039/d5cc02127h

**Published:** 2025-06-09

**Authors:** Sami Malola, Hannu Häkkinen

**Affiliations:** a Department of Physics, Nanoscience Center, University of Jyväskylä FI-40014 Jyväskylä Finland hannu.j.hakkinen@jyu.fi; b Department of Chemistry, Nanoscience Center, University of Jyväskylä FI-40014 Jyväskylä Finland

## Abstract

DFT calculations predict that photophysics of DNA-stabilized silver cluster can be tuned by controlling the oxidation state. The results show that the position and shape of the first absorption band depend dramatically on the total number of delocalized metal electrons in the silver core, varying from a well-defined peak around 755 nm to a broad band between 1100 and 1400 nm by a change of only four electrons. The photophysics can be straightforwadly explained by analysing the frontier orbitals that show a particle-in-a-box character. This predicted sensitivity of the photophysical properties to the oxidation state is relevant for considering the performance of DNA-stabilized silver clusters in NIR-II biological imaging.

## Abstract 2

Density functional theory (DFT) calculations are used to analyse the electronic structure and optical absorption of a DNA-stabilized rod-like Ag_28_Cl_2_ nanocluster that was recently identified from experiments [G. Romolini *et al.*, *Small Struct.*, 2025, 2500022, DOI: 10.1002/sstr.202500022].

## Graphical abstract

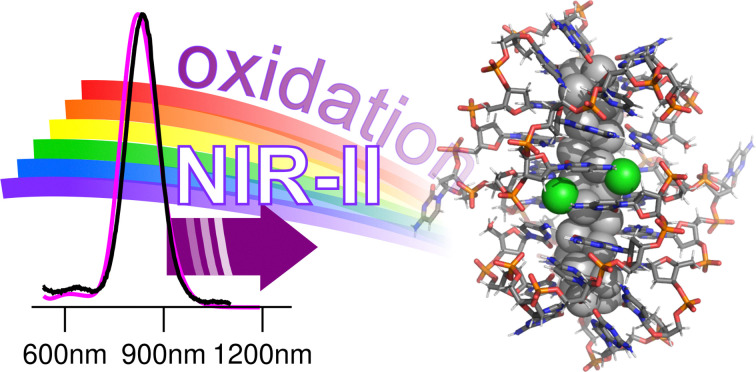

DNA-stabilized silver nanoclusters with 10 to 30 metal atoms are biocompatible by design. They are rather bright and stable emitters, and most intriguingly, their size, shape, and photophysical properties can be tuned by varying DNA's base sequence.^1–12^ Very recently, Romolini *et al.* identified a novel Ag_28_Cl_2_ cluster stabilized by two DNA single strands with a sequence of 5′-CCGCGCGCGCCGCGAA-3′. This cluster has a strong optical absorption at 835 nm and emission at 960 nm with 12% quantum yield in solution.^13^ This work presents a significant advance for these materials to approach the true NIR-II absorption and emission window (roughly 1000–1700 nm) that would enable biological imaging through thicker tissue.

Using the crystal structure (Fig. 1) published in ref. 13, we performed a series of ground-state and linear-response DFT calculations to analyze the electronic structure and optical absorption of this cluster, denoted by 1, and varied the overall charge and chemical composition. It has commonly been observed in previous experiments on DNA-stabilized silver clusters that the metal core is partially oxidized.^4–6,10–12^ The experiment by Romolini *et al.*^13^ determined that the count of metal free electrons in the rod-like Ag_28_Cl_2_ cluster is 12 which implies a nominal [Ag_28_Cl_2_]^+14^ charge. The 16-base DNA strands 5′-CCGCGCGCGCCGCGAA-3′ have 15 phosphate groups each, which we modelled as deprotonated negative units to mimic pH = 7 conditions in water. Furthermore, we found 2 guanine bases in each strand to be in a deprotonated state in the crystal structure data, increasing the total assigned charge for the two DNA strands to be −34. Hence a general formula for the 12-electron system can be written as [DNA]_2_^−34^–[Ag_28_Cl_2_]^+14^ giving the total charge to be used in the DFT calculations as −20. We denote this cluster as 1(12e) in short. We derive three other related systems from this “base” systems as follows. 1(10e) corresponds to a total charge of −18 and 1(14e) a total charge of −22. Additionally, we removed the Cl^−^ ligands from the structure and re-calculated the properties corresponding to the system of 12 free-electrons. We denote that cluster as 2(12e) and it corresponds to a total charge of −18 with a composition of [DNA]_2_^−34^–[Ag_28_]^+16^. These variations are motivated by the known sensitivity of the photophysical properties of small clusters to charge and by the fact that ref. 13 reported that while the chlorido-ligands were identified in the crystal structure, signals from mass spectrometry data in the solution phase did not show the Cl^−^ presence in the clusters. Coordinate files for 1(12e) and 2(12e) are available in the ESI.†

**Fig. 1:**
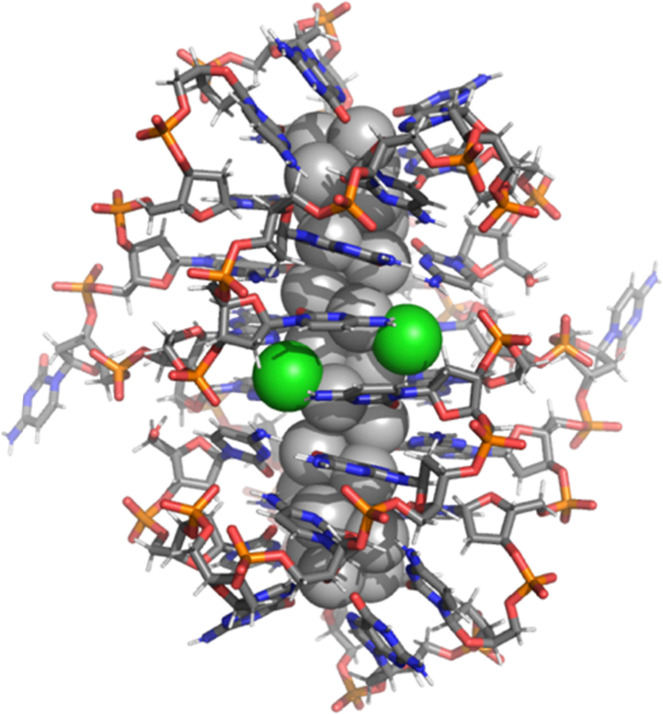
Atomic structure of the (DNA)_2_ – Ag_28_Cl_2_ cluster, stabilized by two DNA strands of 16-base sequence 5′-CCGCGCGCGCCGCGAA-3′ (ref. 13). Ag: grey spheres, Cl: green spheres, C: grey sticks, N: blue sticks, P: orange sticks, and O: red sticks.

All calculations were done by using the GPAW DFT code,^14,15^ and the clusters were solvated by an implicit solvent. We used the GLLB-SC exchange–correlation functional^16^ for the ground-state calculations and the PBE functional^17^ as the kernel in linear-response time-dependent (LR-TDDFT) calculations for optical absorption and circular dichroism spectra. This combination of functionals was shown to work best for smaller DNA-stabilized silver clusters Ag_16_Cl_2_ in our previous study,^11^ judged by comparison of the computed optical spectrum to measured data. We also found in ref. 11 that it is essential to include solvation effects in the calculation due to the highly negatively charged DNA backbone. The methodological choices in the current work are based on this previous experience (see the ESI† file for further computational details).

Fig. 2 shows the comparison of the calculated absorption spectra for 1(10e), 1(12e), 1(14e) and 2(12e), as well as the experimental data reproduced from ref. 13. The results show the excellent agreement between the computed spectrum of 1(12e) and the experimental data, with the peak positions being 821 nm and 835 nm, respectively. This result confirms the existence of the 12 free-electron system in the cluster, as already deduced in the experimental work. It is also worth noting that the computed spectrum of cluster 2(12e) is very similar (except for a minor peak at 600 nm) to that of 1(12e) and experimental data. The similarity of the electronic structures of 1(12e) and 2(12e) is evident also in Fig. 3 that shows the electronic density of states, the energy gap between the highest occupied (HOMO) and lowest unoccupied (LUMO) orbitals as well as real-space visualization of the frontier orbitals. The HOMO–LUMO energy gaps of 1(12e) and 2(12e) are 1.01 eV and 1.02 eV, respectively, and their HOMO states are both particle-in-a-box-like states with 5 node planes. Here we note that the HOMO state of a similar, but shorter DNA-stabilized Ag_16_Cl_2_ cluster^10,11^ has two node planes for a 6-electron system. In fact, all clusters shown in Fig. 3 as well as the Ag_16_Cl_2_ cluster^10,11^ discussed earlier follow the appropriate trend: the HOMO has *N*_e_/2 − 1 node planes for *N*_e_ free electrons in an elongated quantum box. In 1(12e) and 2(12e), the LUMO state has one additional node plane as compared to the HOMO, which defines the character of the lowest-energy optical absorption peak as a dipole-allowed HOMO-to-LUMO transition. This is clearly shown by our analysis in Fig. S1 (ESI†) where the first absorption peak is broken into components of single particle-hole transitions.

**Fig. 2:**
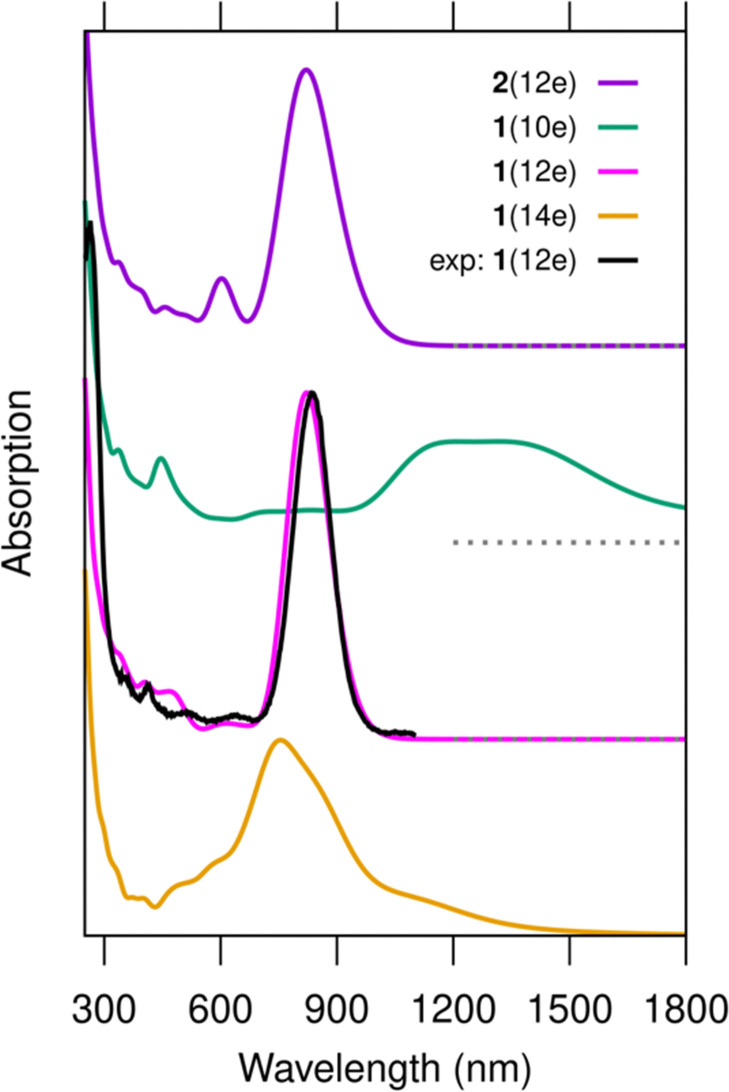
Computed optical absorption spectra of clusters 1(10e), 1(12e), 1(14e) and 2(12e) as compared to the experimental data. The experimental data are reproduced from ref. 13. The peak at 835 nm in the experimental data is scaled to match the intensity of the corresponding peak of cluster 1(12e). No other shifts or scaling in the computed or experimental data are applied. The nominal charge distributions in the clusters can be written as [DNA]_2_^−34^–[Ag_28_Cl_2_]^+*q*^, where *q* = 16, 14, and 12 for 1(10e), 1(12e), and 1(14e), respectively. For cluster 2(12e), it is [DNA]_2_^−34^–[Ag_28_]^+16^. The spectra are calculated using the GLLB-SC functional for ground state wave functions and the PBE functional for the exchange–correlation kernel in the LR-TDDFT calculation. The dashed line below the spectrum of system 1(10e) marks the corresponding zero level.

**Fig. 3:**
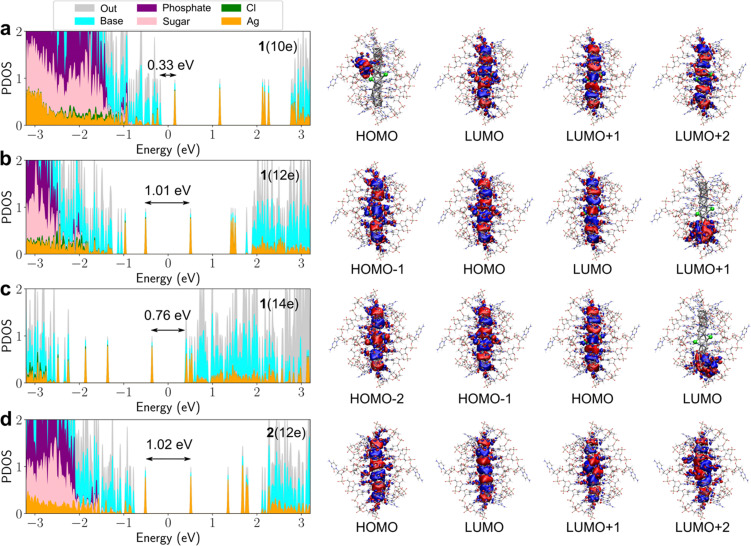
Projected density of electron states PDOS (left) and selected frontier orbitals (right) of (a) 1(10e), (b) 1(12e), (c) 1(14e) and (d) 2(12e). The HOMO–LUMO energy gap is centered around zero energy and its magnitude is shown in the PDOS graphs. The projection is made onto the components of the cluster as shown in the colour label. The data were calculated by using the GLLB-SC functional.

This conceptually simple photophysics applies also for clusters 1(10e) and 1(14e), but with important contributions from ligand states (bases). The HOMO–LUMO energy gap of 1(10e) is only 0.33 eV, with the HOMO state being localized to some of the bases and the LUMO state being virtually identical to the HOMO of 1(12e). There is a high density of occupied states localized on bases just below the HOMO, and these states contribute to the wide absorption band of 1(10e) in the range of 1100–1400 nm (Fig. 2) as shown by the analysis in Fig. S1 (ESI†). The HOMO–LUMO energy gap of 1(14e) is 0.76 eV, with the HOMO being virtually identical to the LUMO of 1(12e) but there is a high density of empty states just above the LUMO, some of them being localized in the silver core and some to the bases. These states make a more collective character and a broad and asymmetric shape of the lowest-energy absorption peak at 755 nm (Fig. S1, ESI†).

Based on the results from Romolini *et al.*^13^ the Ag_28_Cl_2_ cluster can exist in solution with or without the chloride ligands, and the similarity of the absorption spectra of 1(12e) and 2(12e) indirectly supports this observation. However, our work predicts that there are significant differences in the chiral optical response of 1(12e) and 2(12e). In the computed chiral dichroism (CD) spectra, the removal of the chlorido ligands (cluster 2(12e)) dramatically changes the intensity and sign of the CD signal at 821 nm (Fig. 4). The analysis of the rotational strengths of this transition (Fig. S2, ESI†) shows that the absence/presence of the chlorido ligands has an indirect effect on the rotational contributions of a number of electronic states in a broad energy window. Particularly, the presence of Cl^−^ creates a strong negative contribution from the HOMO–LUMO transition in 1(12e) (Fig. S2a, ESI†).

**Fig. 4:**
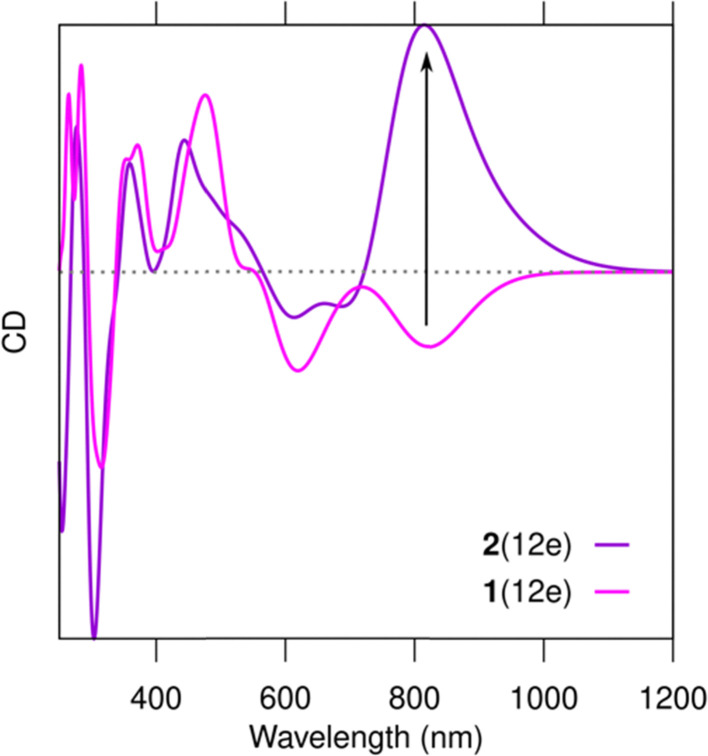
Computed CD spectra of clusters 1(12e) and 2(12e). Computational choices for spectra and wave functions as in Fig. 2.

While the sensitivity of the computed absorption properties of Ag_28_Cl_2_ to the free-electron count, and indirectly to the total oxidation state of the system, may seem surprising, it can be straightforwardly understood from our DFT analysis indicating rather simple photophysics among electron states that highly resemble quantum mechanical particle-in-a-box model. It has been previously emphasized that a higher aspect ratio of elongated metal-based clusters should tune absorption (and emission) close to the second infrared window NIR-II.^18^ Overcoming the challenges to synthesize even longer rod-like DNA-stabilized silver clusters would undoubtedly push the photophysics eventually to NIR-II. However, this work implies another potential chemical route to tune the photophysics, if the total oxidation state of the DNA-stabilized cluster could be controlled. A more oxidized state of the cluster pushes the photophysics to the NIR-II window. This is qualitatively in agreement with earlier experimental data from the Copp group.^19^ Finally, one should note that the local pH environment in a biological system may vary considerably, which may change the oxidation state of the emitter in operation, changing also its photophysical behaviour.

This work was supported by the Research Council of Finland (grant 355083) and by the European Union (ERC AdvG project DYNANOINT). The computations were made at the Finnish national supercomputing center CSC (computing grant 2002721).

## Conflicts of interest

There are no conflicts to declare.

## Supplementary Material

CC-061-D5CC02127H-s001

CC-061-D5CC02127H-s002

CC-061-D5CC02127H-s003

## Data Availability

The data supporting this article have been included as part of the ESI.†
