# Supplementary material for: Tuning the electronic structure of a rod-like DNA-stabilized silver nanocluster Ag28Cl2 for photophysics in the NIR-II window
Source: Chem Commun (Camb). 2025 Jun 9;61(57):10598–601. doi: 10.1039/d5cc02127h (PMC12172426; doi:10.1039/d5cc02127h)
Supplement: CC-061-D5CC02127H-s003 [file CC-061-D5CC02127H-s003.pdf]

Supplementary Information (SI) for ChemComm.
This journal is © The Royal Society of Chemistry 2025

Electronic Supplementary Information

Tuning the electronic structure of a rod-like DNA-stabilized silver nanocluster $Ag_{28}Cl_2$ for photophysics in the NIR-II window

Sami Malola and Hannu Häkkinen

Computational methods

The calculations were done using density functional theory as implemented in code package GPAW.[1,2] Real-space grid and implicit solvent model of water was used in calculations with 0.3 Å grid spacing and GLLB-SC exchange correlation functional.[3] The crystal structure published by Romolini et.al. [4] was used as the starting point (with water molecules found in the data removed and hydrogen atoms added in chemically relevant sites using PyMol) in the calculations of clusters **1**(10e), **1**(12e) and **1**(14e), from which **1**(12e) corresponds to the measured cluster. The total charges of the calculated clusters were -18, -20 and -22 respectively. The structure of the model cluster **2**(12e) was otherwise the same but Cl-atoms were removed. Total charge for **2**(12e) was -18. The total charges were fixed based on the measured cluster **1**(12e) so that the superatom electron count matches the experimentally determined 12 electrons. The selected total charge takes into account that all the phosphate groups have negative -1 charge and that there are two deprotonated bases per strand. These two deprotonated guanines in each DNA-strand are located very close to the Ag-core in the resolved experimental structure and do not allow addition of the proton as expected in their neutral charge state. Implicit solvent model was used as parametrized in ref. [5].

The localization of the electron states was analysed by projecting the density of states to atoms. Projection was done first to spherical harmonics functions centered at each atom using cutoff radii Ag: 1.5 Å, Cl: 1.3 Å, P:0.8 Å, O: 0.7 Å, C: 0.7 Å, N: 0.7 Å, H: 0.5 Å.[6] The summation over the atoms in the atomic groups and over the angular momenta was taken to get the total weights for Ag- and Cl-atoms and for bases, sugar and phosphate groups of the DNA strands. Linear response time-dependent density functional theory (LR-TDDFT) was used to calculate the optical absorption and the CD spectra.[7] PBE functional [8] was used as a xc-kernel in LR-TDDFT calculation, since GLLB-SC does not provide density gradients and is thus not suitable to be used in the kernel. This combination of functionals (GLLB-SC for ground state wave functions, PBE for LR xc kernel) was motivated by our previous work on similar but smaller DNA-stabilized silver clusters (ref. 11 in the main text).

The origins of the features of the absorption and of the CD spectra were analysed with dipole and rotatory strength transition contribution maps (DTCM & RTCM) using time dependent density functional perturbation theory.[9] The results were reported as a total contributions combining

the analysis results from all three cartesian coordinate directions of the polarization of the weak perturbative external electromagnetic field. These directions were selected based on the principal axis of moments of inertia of the cluster. The map shows as a contour plot the strengthening and screening contributions at the selected energy/wavelength decomposing them to excitations between Kohn Sham states.

References

[1] Mortensen J. J. et. al. GPAW: An open Python package for electronic structure calculations, *J. Chem. Phys.* **2024**, 160, 092503

[2] Enkovaara, J. et al. Electronic structure calculations with GPAW: a real-space implementation of the projector augmented-wave method, *J. Phys. Condens. Matter* **2010**, *22*, 253202.

[3] Kuisma, M.; Ojanen, J.; Enkovaara, J.; Rantala, T. T. Kohn-Sham potential with discontinuity for band gap materials. *Phys. Rev. B* **2010**, 82, 115106.

[4] G. Romolini et. al. Shining Bright at 960 nm: A 28-Silver-Atom Nanorod Stabilized by DNA, *Small Struct.* **2025**, 2500022

[5] Held A.; Walter M., Simplified continuum solvent model with a smooth cavity based on volumetric data, *J. Chem. Phys.* **2014**, 141, 174108.

[6] Walter, M.; Akola, J.; Lopez-Acevedo, O.; Jadzinsky, P. D.; Calero, G.; Ackerson, C. J.; Whetten, R. L.; Grönbeck, H.; Häkkinen, H. A unifiedview of ligand-protected gold clusters as superatom complexes, *Proc. Natl. Acad. Sci. U.S.A.* **2008**, *105(27)*, 9157–9162.

[7] Walter, M. et al. Time-dependent density-functional theory in the projector augmented-wave method. *J. Chem. Phys.* **2008**, *128*, 244101.

[8] Perdew, J. P.; Burke, K.; Ernzerhof, M. Generalized gradient approximation made simple. *Phys. Rev. Lett.* **1996**, *77*, 3865–3868.

[9] S. Malola, L. Lehtovaara, J. Enkovaara, H. Häkkinen, Birth of the Localized Surface Plasmon Resonance in Monolayer-Protected Gold Nanoclusters, *ACS Nano* **2013**, *7*, 10263-10270.

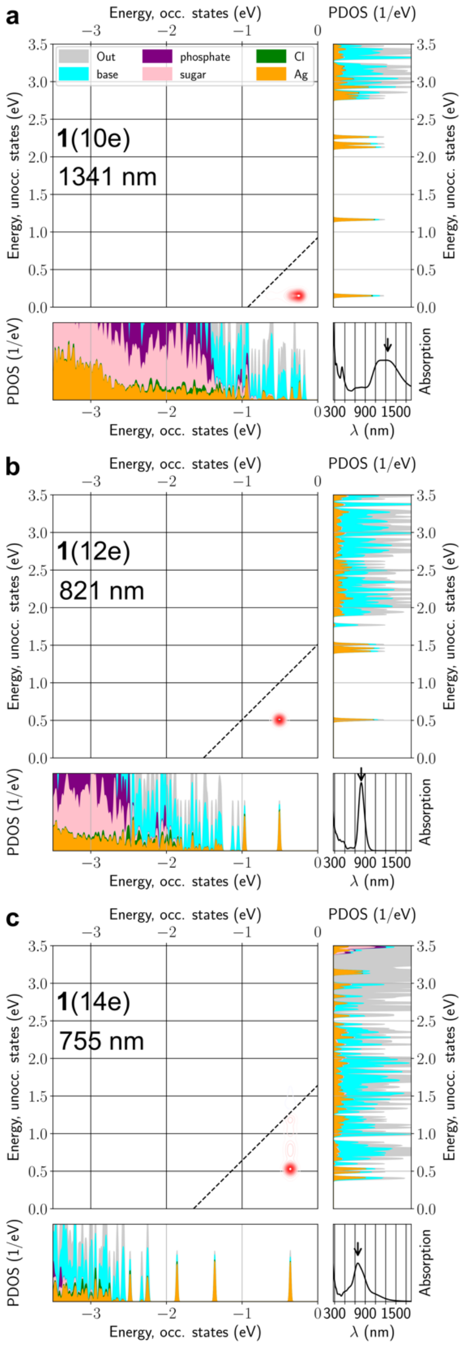

Figure S1. Dipole transition contribution maps for the lowest energy absorption peak. Along the dashed line, the energy difference of independent one electron – one hole orbitals would match the absorption energy.

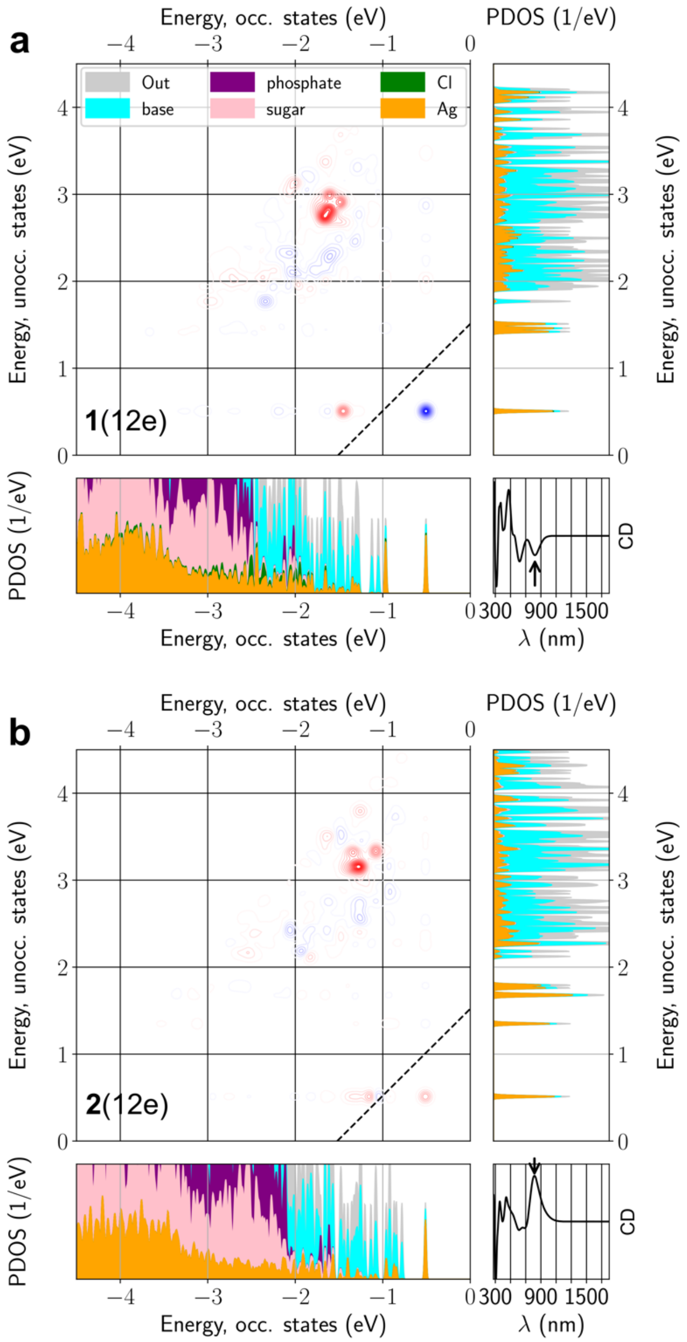

Figure S2. Rotatory strength contribution maps for the lowest energy CD signal. Red and blue contributions denote positive and negative contributions, respectively. Along the dashed line, the energy difference of independent one electron – one hole orbitals would match the absorption energy.
